# Interstitial lung disease and the STING pathway

**DOI:** 10.1172/JCI204544

**Published:** 2026-06-01

**Authors:** Prasad Palani Velu, Gaofeng Zhu, Karen J. Mackenzie

**Affiliations:** Centre for Inflammation Research, Institute for Regeneration and Repair, University of Edinburgh, Edinburgh, United Kingdom.

## Abstract

Identification of the genetic mutations underlying the ultrarare monogenic conditions STING-associated vasculopathy with onset in infancy (SAVI) and coatomer protein complex subunit alpha (COPA) syndrome revealed a role for the stimulator of interferon genes (STING) immune pathway in the pathogenesis of interstitial lung disease (ILD) in these conditions. STING-focused therapeutics could be a potential avenue for the treatment of SAVI and COPA syndrome in the future, yet the relevance of STING to more common types of ILD is not clear. Here, we provide an overview of SAVI and COPA syndrome, the nature of ILD in these conditions, and current evidence regarding STING activity in their pathogenesis. We discuss data from studies of a variety of other ILDs and model systems and explore the potential role for STING in more common forms of ILD.

## Introduction

There is intense interest in the role of the DNA sensing pathway cGAS/STING (cyclic GMP-AMP synthase [cGAMP]/stimulator of interferon genes) in inflammatory disease (1). Gain-of-function (GOF) mutations in *STING1*, the gene encoding STING, cause the monogenic interferonopathy STING-associated vasculopathy with onset in infancy (SAVI), in which interstitial lung disease (ILD) is a predominant feature (2). This, together with mechanistic evidence of enhanced STING activity in the more recently described coatomer protein complex subunit alpha (COPA) syndrome (3–5), directly implicates STING activity in the pathogenesis of ILD in these ultrarare genetic disorders. The study of rare, monogenic diseases is vital for patients affected by these diseases and is a proven means of gaining insight into the pathogenesis of more complex, multifactorial diseases (6).

Notably, many of the current paradigms for the initiation of more common types of ILD, for example, idiopathic pulmonary fibrosis (IPF) and chronic hypersensitivity pneumonitis (CHP), imply the involvement of processes such as DNA damage (7), which have the potential to trigger innate immune signaling via the cGAS/STING pathway (8). However, the relationship of STING to more common types of ILD is not yet fully understood.

Here, we describe the current understanding of how hyperactivation of the STING pathway occurs in SAVI and COPA syndrome and its role in the pathogenesis of those conditions. Outlining the existing knowledge about the role of the STING pathway in ILD found in SAVI and COPA syndrome enabled us to extend to a wider discussion about the potential role of STING in other forms of ILD. We propose that greater understanding of STING biology in the lung could aid the development of novel therapeutic approaches for both rare and more common forms of ILD.

## What is ILD?

ILD and childhood ILD (chILD) are diverse groups of pulmonary disorders characterized by inflammation and/or fibrosis within the alveolar interstitium, the space between the alveolar epithelium and capillary endothelium (9–11). The alveolar interstitium comprises lymphatics, scant fibroblasts, and ECM proteins that maintain alveolar acinus structure and facilitate gas exchange (11). Inflammation and fibrosis within this space lead to impaired gas exchange and tissue distortion and ultimately manifest in respiratory failure (9, 11).

Adult ILDs are classified predominantly based on etiology, with each entity possessing distinct radiological and histopathological characteristics (12). In adults, ILD diagnoses include idiopathic interstitial pneumonias (of which IPF is the most common, affecting 0.33–4.51 per 10,000 people worldwide; ref. 13), CHP, connective tissue disease–associated ILD (CTD-ILD), drug-induced ILD, occupational ILD (e.g., silicosis), and postinfection ILD (reviewed in ref. 9). Adult ILDs can additionally be categorized by disease behavior. A label of progressive pulmonary fibrosis is agnostic to the underlying condition but constitutes worsening respiratory symptoms with declining physiological or radiological features indicative of progressive fibrotic change (14). chILD is thought to be around 10-fold rarer than adult ILD and includes distinct etiologies (10). chILDs are categorized into ILDs that typically manifest in early childhood and those without a specific relationship to age (10, 15). Different forms of ILD and chILD have distinct radiological appearances on thoracic high-resolution computed tomography (HRCT) scans, which correlate well with underlying histopathological alterations (10, 14).

The investigation of chILD and adult ILDs in parallel may yield several benefits. From a clinical perspective, individuals with nonlethal chILDs will transition to adult services, requiring ongoing care from clinicians trained in the management of adult ILDs. Here, a working knowledge of both chILD and adult ILDs would prove beneficial. In addition, the clearly defined genetic basis of certain chILDs can help parse the contribution of specific biological pathways to the pathogenesis of many ILDs for which etiology and factors affecting progression are less well understood.

### ILD: a spectrum of fibrosis and inflammation.

IPF is the archetypal fibrotic ILD. While the exact pathogenic processes underlying IPF remain uncertain, varying contributions from genetic susceptibility, aging, and environmental exposures are thought to result in epithelial dysfunction as an early initiating feature (16, 17). An aberrant wound-healing response, with failure to effectively reconstitute alveolar epithelium and the emergence of aberrant epithelial cell populations, has been described (18, 19). Concurrent changes in phenotypes and frequencies of immune and stromal cell populations promote excessive deposition of ECM (18–21). Although the etiology of fibrotic lung diseases may vary, there is considerable overlap in the downstream pathways that promote fibrogenesis (11, 14, 22, 23). The role of inflammation in the pathogenesis of fibrotic ILDs such as IPF remains unclear, as the use of immunomodulatory therapies has yielded no clinical benefit and instead increased adverse events (24). These observations have led some to surmise that inflammatory changes in IPF may occur independently of, or precede, the progressive fibrotic remodeling seen at clinical presentation of IPF (25).

However, inflammation is a prominent feature of several ILDs, including CTD-ILD and CHP (26). In these conditions, dysregulated inflammation is thought to lead to progressive lung damage, fibroblast activation, and subsequent fibrosis (11, 22). Response to corticosteroids or other antiinflammatory agents in these ILDs is an indicator of underlying inflammation (11, 26, 27). HRCT features indicative of inflammation include ground glass opacification (GGO), a nonspecific interstitial pneumonia pattern, and the absence of features classically associated with fibrotic ILD (honeycombing, traction bronchiectasis, and peripheral and basal predominance) (26, 27). Histopathology from inflammatory ILDs features extensive cellular inflammation and can include hallmarks such as lymphoid follicle and/or granuloma formation (27).

### Genetics and ILD.

**Figure 1:**
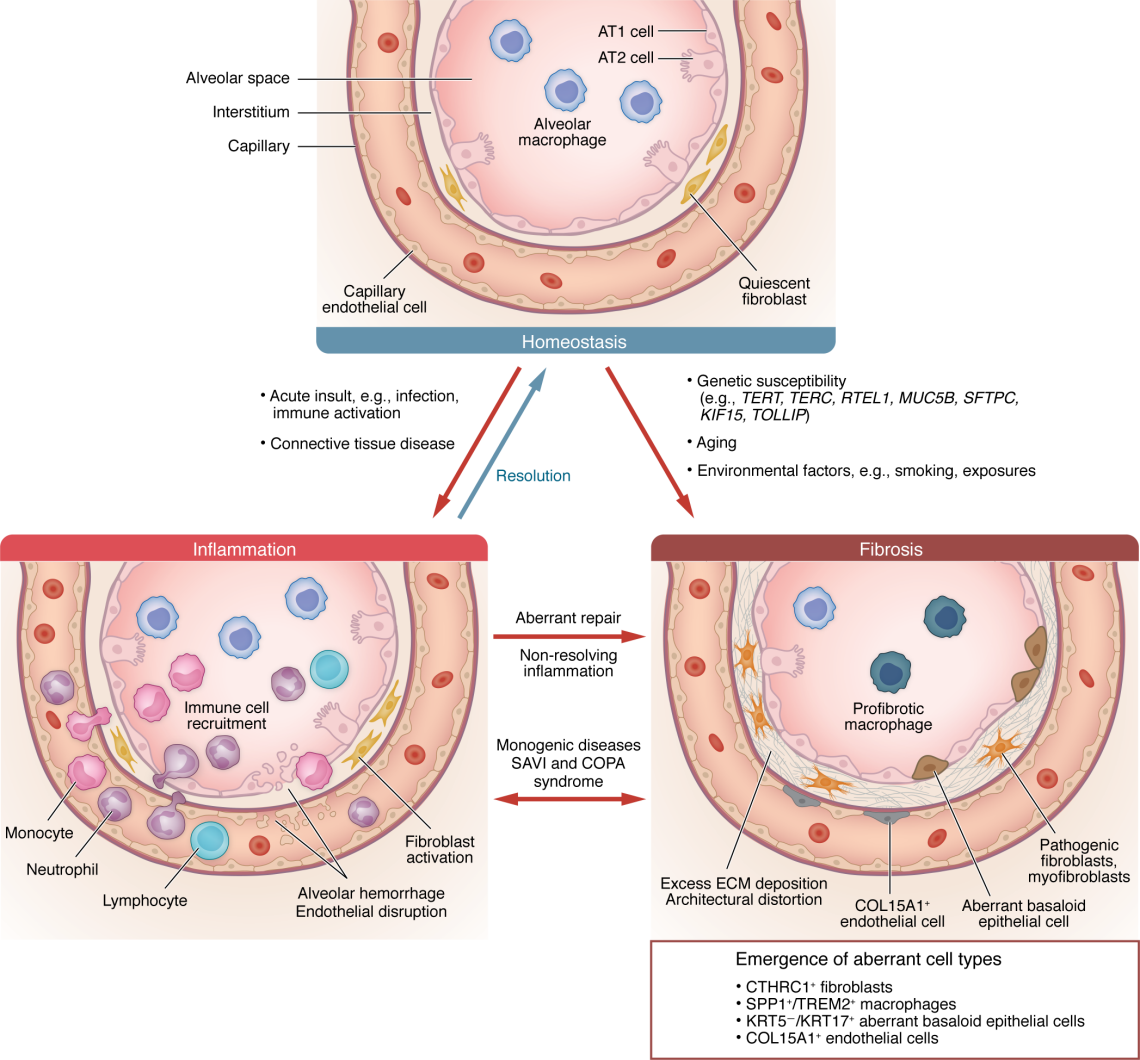
A conceptual framework for ILD pathogenesis. Predisposition to ILDs is influenced by genetic factors (variants associated with increased risk of ILD or rare germline mutations causing ILD), environmental exposures (e.g., smoking, dust), acute lung injury (e.g., infection), existing connective tissue disease, and aging. The propensity toward inflammatory and/or fibrotic phenotypes varies depending on type and stage of ILD. Resolution of acute insult can result in return to homeostatic conditions, or chronic inflammation or aberrant repair can lead to the establishment of fibrosis. In addition, IPF is thought to arise without any preceding inflammation. SAVI and COPA syndrome variably feature concurrent inflammation and fibrosis. AT1, type 1 alveolar; AT2, type 2 alveolar; TERT, telomerase reverse transcriptase; TERC, telomerase RNA component; RTEL1, regulator of telomere elongation helicase 1; SFTPC, surfactant protein C; KIF15, kinesin family member 15; TOLLIP, toll interacting protein.

Family history is the strongest risk factor for IPF (28), and many genetic variants, including those in genes relating to surfactant, telomerase, mucin-5B, and certain immune responses, have been associated with an increased risk of developing ILD (28; reviewed in ref. 29). These genetic susceptibility variants contribute to a complex interplay with other risk factors for ILD, including environmental exposures such as cigarette smoking (16). Monogenic variants underlie certain cases of rare familial ILD and account for 20%–30% of chILD (10). Variants in certain genes may also be associated with other characteristic clinical presentations (refer to ref. 15 for a comprehensive review). Figure 1 depicts the pathogenic features and risk factors across the ILD spectrum.

Studying the genes affected in monogenic forms of ILD can help identify the biological processes involved in ILD pathogenesis and could inform novel therapeutic approaches. Several of the single-gene mutations implicated in chILD have been investigated using murine models, providing potential insights into how these mutations may cause ILD (reviewed in refs. 30, 31). For example, mutations in proteins involved in surfactant production, including *Abca3*, *Sftpa1*, and *Sfptc*, induced inflammation and fibrotic responses in mice (30, 32, 33). Conditional deletion of *Trf2* in mouse type 2 alveolar epithelial cells (to induce telomere dysfunction in these cells) resulted in epithelial senescence, immune cell recruitment, and increased mortality following bleomycin instillation (34). Polymorphisms in some of these genes have also been identified in familial pulmonary fibrosis (PF) and in IPF GWAS analyses, where background genetic susceptibility appears to synergize with exposure to environmental factors (e.g., smoking, inhaled dust) to promote the development of ILD (35). Table 1 outlines our current understanding about genes involved in the pathogenesis of different types of ILDs.

Recently, the identification of germline mutations causing the monogenic ILDs SAVI and COPA syndrome have implicated the STING pathway in ILD (2–5, 36). This has raised interest in how aberrant STING activity can lead to ILD and the potential relevance to other forms of nonsyndromic fibrotic lung disease.

## The cGAS/STING pathway

A cell’s ability to sense and respond to non-self DNA is vital to defend against pathogens. STING is a transmembrane protein first described as an activator of innate immune responses, particularly type I IFN (IFN-I) production and antiviral immune responses (37).

STING can bind bacterium-derived cyclic dinucleotides, such as cyclic di-GMP, to directly initiate an immune response (38). In 2013, the Chen group discovered that STING could also become activated by the second messenger cGAMP (39), which was synthesized through the catalytic activity of an enzyme called cGAS, activated upon DNA binding (40). cGAS is now known to be a key intracellular sensor for dsDNA (reviewed in ref. 41). Both cGAS and STING are widely expressed across different cell types, including parenchymal and immune cells in the lung (42).

cGAS binds dsDNA without sequence constraints, meaning that in addition to its role in recognizing pathogen-derived DNA, cGAS also has the potential to respond to host DNA (43). cGAS primarily resides within the nucleus, where its activity is tightly regulated through various mechanisms to avoid aberrant activation by host genomic DNA (44, 45). However, it is also found in the cytosol, enabling swift detection of invading pathogens (40). Alterations to the compartmentalization or integrity of host genetic material arising as a result of processes such as genome instability, cell death, or mitochondrial stress, have been shown to trigger a cGAS-driven immune cascade (reviewed in ref. 8).

**Figure 2:**
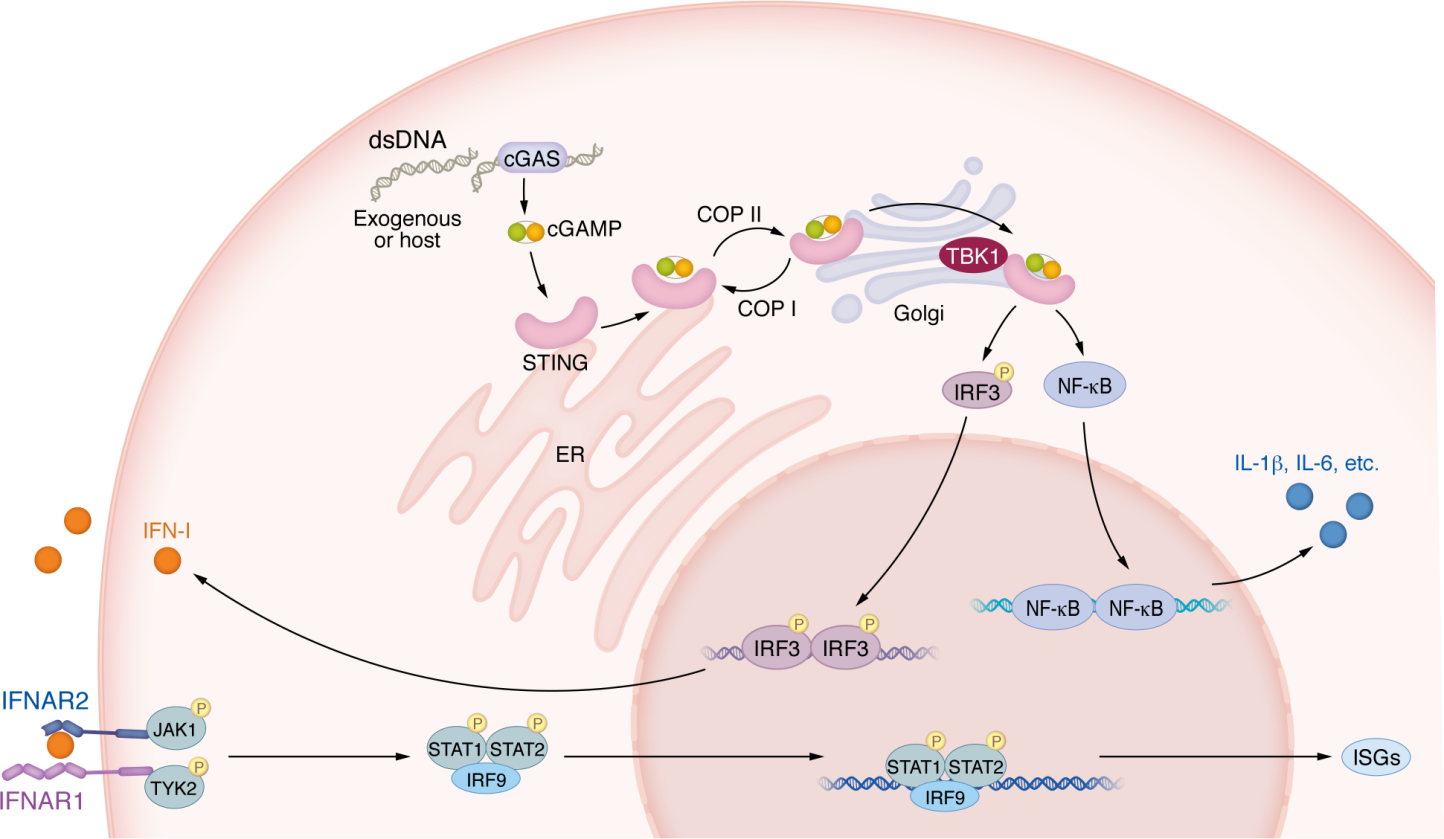
Immune responses following dsDNA sensing by cGAS/STING. dsDNA derived from pathogens (e.g., viruses, bacteria) or from the host itself (e.g., as a consequence of DNA damage) binds to cGAS, leading to the generation of cGAMP. cGAMP binds STING on the ER, leading to a conformational change and trafficking to the Golgi, facilitated by COP-II. In the Golgi, STING recruits TBK1, initiating a downstream signaling cascade via IRF3 and NF-κB, resulting in the production of IFN-I, ISGs, and proinflammatory cytokines such as IL-6. Autocrine and/or paracrine activity of IFN-I generates further ISGs. COP-I mediates retrograde transport of STING from the Golgi to the ER. IFNAR1, interferon alpha and beta receptor subunit 1; IFNAR2, interferon alpha and beta receptor subunit 2; TYK2, tyrosine kinase 2.

Binding of cGAMP to STING causes a conformational change in STING and induces its translocation from the ER to the Golgi, via the ER-Golgi intermediate compartment (ERGIC) vesicles (8). While in the Golgi, STING recruits TANK-binding kinase 1 (TBK1), which in turn becomes activated through autophosphorylation (46). Activated TBK1 then phosphorylates residues in a region of the C-terminus of STING (Ser366) (47), enabling the recruitment of the transcription factor IFN regulatory factor 3 (IRF3). IRF3 is then itself phosphorylated by TBK1 (47, 48), leading to its dimerization and translocation to the nucleus to induce expression of IFN-I. STING activation also initiates a proinflammatory immune response via NF-κB, generating cytokines such as IL-6 and IL-1β (49, 50) (Figure 2).

**Figure 3:**
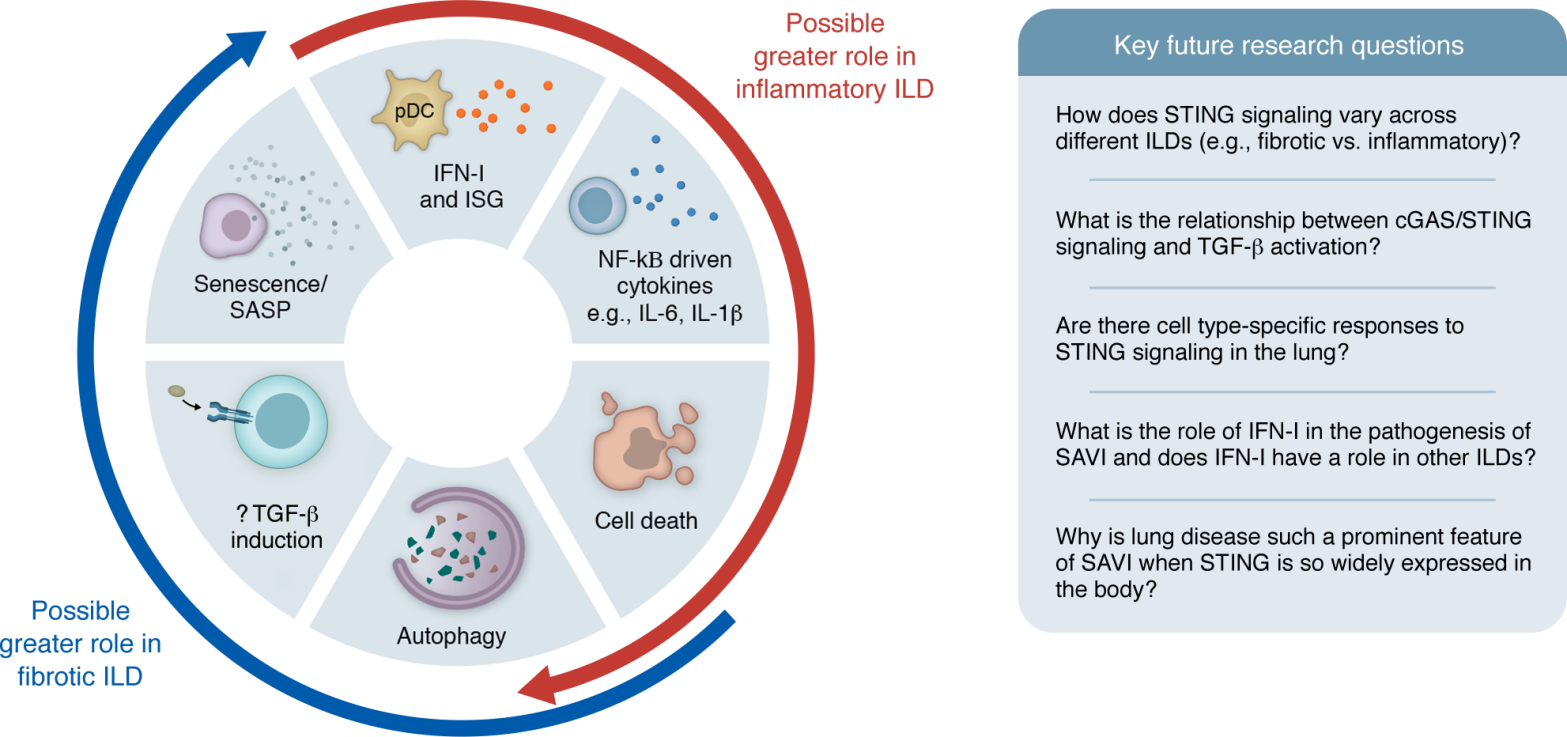
Potential outcomes of STING pathway activation. The varied consequences of STING pathway activation continue to be ascertained. In addition to its prominent role in the production of IFN-I and other proinflammatory mediators, STING activation has also been shown to have roles in inducing cell death, autophagy, and senescence responses. Biological outcomes are likely to vary depending on the nature of STING activation, and this remains an active area of study. Here, we postulate that some outcomes from STING signaling could be more pertinent to types of ILD that have strong fibrotic components compared with ILDs where robust inflammatory phenotypes are more evident. However, interplay between the different outcomes of STING signaling is likely, and the role of inflammation and fibrosis in some ILDs may also be indistinct. Important questions for future research are highlighted. SASP, senescence-associated secretory phenotype.

More recently, roles for STING in other processes, such as autophagy (51, 52), senescence (53), lysosomal biogenesis (54), and apoptosis (55), have been described (Figure 3). STING-driven immune responses can also occur independently of cGAS in some contexts (56). TGF-β, often a key mediator in fibrosis, has been shown to be produced by T cells following cGAS/STING stimulation (57). However, suppression of STING responses by TGF-β has also been shown (58), and the relationship of TGF-β to STING signaling requires further study (Figure 3).

Interestingly, the immune response induced by cGAS/STING in different cell types has been noted to vary, in part thought to relate to differences in baseline STING expression and signal strength variation (55, 59). The role of cGAS-dependent and -independent STING activation in different disease settings is an intriguing area of study. It is possible that different outcomes from STING activation may be more pertinent to certain types of ILD, for example, those with a strong fibrotic element, compared with ILDs where inflammation is a prominent feature (Figure 3).

## SAVI and COPA syndrome

The monogenic conditions SAVI and COPA syndrome are associated with elevated IFN-stimulated gene (ISG) expression in the blood (4, 60) and as such are recognized as type I interferonopathies (T1Is) (36, 61). Unlike the archetype T1I Aicardi-Goutières syndrome, where the principal features are neurological, patients with SAVI or COPA syndrome usually do not develop central nervous system disease; rather, pulmonary involvement is the most common feature of these disorders (36, 61). Table 2 outlines the nature of the mutations that can cause SAVI and COPA syndrome.

### SAVI inheritance and clinical features.

First described in 2014, SAVI is caused by GOF mutations in *STING1* (2), and fewer than 100 individuals with SAVI have been described in the literature to date (62). Inheritance can be autosomal dominant, but more often, heterozygous mutations are acquired de novo (61, 63). Homozygous mutations causing SAVI, albeit even rarer, have also been documented (64).

SAVI-associated mutations correspond with almost complete penetrance of disease, and, sadly, mortality is high (2, 61). The age of onset of clinical features is typically young, often under 1 year old, and around 80% of patients with SAVI develop ILD, though the degree of lung involvement is highly variable (61, 63, 64). Cutaneous vasculopathy, telangiectasias, and distal tissue damage are also frequently seen (63, 65). HRCT features in SAVI can vary considerably but are largely suggestive of an inflammatory etiology, with key findings including GGO, interspersed with interlobular and intralobular septal thickening resulting from edematous/inflamed interstitium (a radiological feature termed “crazy paving”) and cysts (63). These changes often occur in an asymmetric pattern, in contrast to CTD-ILD (61). Radiological intrathoracic lymphadenopathy can also exist (63). Histopathological analyses of lung biopsies from affected individuals demonstrate mixed lymphocytic infiltrate and interstitial fibrosis (62), and analysis of bronchoalveolar lavage (BAL) fluid has shown varying proportions of lymphocytes, neutrophils, and/or hemosiderin-laden macrophages (66).

SAVI is associated with early progression to pulmonary fibrosis, with radiological fibrosis evident in as many as 50% of patients with lung involvement, even at an early age (61, 62). Antineutrophil cytoplasmic antibodies are relatively common, whereas anti-dsDNA antibodies are not (63). Other inflammatory conditions, including arthritis, may also occur, and inflammatory markers are usually elevated (61, 63).

### SAVI and the STING pathway.

SAVI-associated GOF mutations affect STING localization, causing cGAS- and cGAMP-independent Golgi retention, resulting in reduced degradation and chronic activation of STING (2, 46, 67, 68). Pathogenic SAVI mutations thus demonstrate a clear link between hyperactivity of STING and this ultrarare ILD.

Individuals with SAVI typically show elevated ISG, TNF-α, and IL-6 expression in PBMCs (2, 60, 63). scRNA-seq of PBMCs from patients with SAVI revealed increased expression of genes related to the integrated stress response, with ISG overexpression most marked in monocytes and DCs (69). A T cell lymphopenia (usually mild) is often reported and is associated with increased frequency of naive T cells and reduced effector and memory T cell populations (63, 69). These altered T cell subsets show strong activation phenotypes and are prone to senescence and cell death (69).

Mice with *Sting1* mutations known to cause SAVI can develop pathologies similar to those found in people with SAVI, including pulmonary inflammation, cutaneous ulceration, and lymphopenia (70, 71). Some mouse models also develop lung fibrosis (71), although no model completely recapitulates all the features of the human disease, and ISG expression in SAVI mouse models is relatively low compared with that seen in human patients (61), adding to existing challenges of directly correlating data from mouse models of the T1Is to the human diseases (36).

Mice with SAVI-associated *Sting1* mutations [p.(N135S) and p.(V154M)] have been found to develop lung disease independently of cGAS or IFN-I (70, 71). How this independence from IFN-I relates to human disease is an intriguing question; it implicates non–IFN-I pathways (such as NF-κB) in this setting of chronic STING activation, an area of active study. However, these data should be interpreted in the context of species-specific differences such as the typically low ISG expression in mouse models (61) compared with the markedly elevated levels seen in patients with SAVI (63). Mice with SAVI-associated *Sting1* mutations also differ in that they can manifest defective lymph node organogenesis, presenting as a SCID phenotype (72, 73); this contrasts with the tendency toward lymphadenopathy seen in people with SAVI (62). JAK inhibitors, which inhibit IFN-I signaling, have shown some therapeutic efficacy in SAVI and some other type I interferonopathies (62, 74), and *Ifnar1* was also found to be required for a vasculopathy phenotype in a SAVI mouse model, where hematopoietic cells were induced to express human STING (75), although those mice did not develop lung inflammation (as discussed further below). The role of IFN-I in SAVI pathogenesis in humans is therefore an area warranting future research (Figure 3).

A role for T cells in inducing lung disease in mice with SAVI mutations has been found (76, 77). Interestingly, nonhematopoietic cells expressing the p.(V154M) SAVI mutation have been shown to recruit WT T cells to the lung to promote inflammation (77, 78). Additionally, one study found that restricted expression of the *Sting1* p.(N154S) SAVI mutation to hematopoietic cells alone was not sufficient to induce lung disease, although a vasculopathy was evident (75). Conditional expression of the *Sting1* p.(V154M) SAVI mutation in endothelial cells was found to induce an influx of immune cells to the lung, although lung inflammation did not reach the level seen in animals with germline SAVI mutations. This indicates that other cell types expressing SAVI mutations play a role in the lung pathology (79). Taken together, these studies suggest roles for both T cells and nonhematopoietic lung cells in the pathogenesis of lung disease in these mouse models of SAVI. The reasons why lung disease is such a prominent feature of SAVI, despite STING being widely expressed throughout the body, remain unclear. The high protein expression of STING in the respiratory system compared with other systems such as the gastrointestinal tract seems relevant (42), with scRNA-seq data revealing high expression of *STING1* in many respiratory-relevant lung types, including stromal and immune cells (42). It is possible that specific respiratory exposures to environmental triggers play a role or that respiratory-specific effects of STING activity are involved. Important questions regarding STING biology in the lung therefore remain (Figure 3).

*STING1* is known to often exhibit genetic variation, and the common human *STING1* haplotype HAQ (R71H-G230A-R293Q) has been found to be hypomorphic (80) and to inhibit the constitutive activation of STING in cells with SAVI-associated mutations (81) (Table 2). The presence of the HAQ haplotype does not prevent SAVI, since an individual with SAVI and the HAQ haplotype has been described, although some amelioration of the phenotype was proposed in that study (82). HAQ and the AQ (G230A-R293Q) haplotype were found to reduce (in the case of HAQ) or prevent (in the case of AQ) disease in a p.(N135S) SAVI mouse model (83). These beneficial effects were associated with increased Treg cells and reduced T cell death, and in vitro studies showed a protective effect of HAQ on STING-induced cell death (83). Further studies into the role of haplotypes such as HAQ and the effects on SAVI phenotypes will be helpful to expand this area of knowledge.

## COPA syndrome: inheritance and clinical features

COPA syndrome was first described in 2015 and is caused by heterozygous missense mutations in the *COPA* gene on chromosome 1 at position 1q23.2 (5). COPA syndrome displays an autosomal dominant mode of inheritance but a notable degree of clinical nonpenetrance, with 17% of *COPA* mutation–positive individuals found to be asymptomatic in a recent comprehensive study (74). De novo and somatic mosaic *COPA* mutations have also been shown to cause COPA syndrome (74).

Around 70 individuals with COPA syndrome have so far been described in the literature, and while disease onset often occurs in infancy or early childhood, clinical features may develop for the first time in adulthood (61, 74, 84, 85). Lung disease is the most frequent feature of COPA syndrome (74). Approximately 80% of individuals with COPA syndrome develop ILD, and diffuse alveolar hemorrhage (DAH) is also prominent (5, 61, 74, 86). Recurrent DAH without externalization may present with features suggestive of recurrent respiratory tract infection, and HRCT appearances can normalize between DAH episodes (61). Radiologic features of ILD in affected individuals include GGO, cysts, nodules, and fibrosis (87, 88). Although lung fibrosis is less common in COPA syndrome than in SAVI (61), a recent study found 37% of people with COPA syndrome had lung fibrosis (74). Fibrosis may arise following follicular bronchiolitis in some cases (86). Histopathological findings in COPA syndrome include nonspecific features of a fibrosing ILD and/or DAH (87) with or without interstitial lymphocytic infiltration (5). Lymphoid follicles and macrophage infiltration into the alveoli are often reported (4, 84, 87). BAL fluid analysis can be consistent with DAH (macroscopically bloody or containing hemosiderin-laden macrophages) or indicative of a nonspecific alveolitis (61).

Clinical presentations affecting other body systems have also been documented. Arthritis is frequently noted in COPA syndrome, typically of a rheumatoid factor–positive polyarticular type (5, 61, 84). Antineutrophil cytoplasmic antibodies are commonly detected, while autoantibodies to dsDNA are notably less frequent, and inflammatory markers, particularly C-reactive protein, do not seem to consistently correlate with disease severity (61, 74). Vascular skin features are less frequent than found in SAVI but are still notable, affecting 32% of people with COPA syndrome in a recent study (74). Renal involvement, such as glomerulonephritis, can be a feature of COPA syndrome (5, 61, 84), and gastrointestinal, cardiac, hepatic, neurological, and other sites have also been reported to be affected to varying degrees in some individuals with COPA syndrome (74).

### COPA syndrome and the STING pathway.

*COPA* encodes the α subunit of the coat protein complex I (COP-I), which mediates the retrograde transport of cellular cargo from the Golgi complex and the ERGIC to the ER (89). COP-I is also involved in intra-Golgi transport (90) and autophagy (91, 92) and has been implicated in the anterograde transport of cargo from the ERGIC to the Golgi (93).

Pathogenic COPA mutations causing COPA syndrome have been shown to reduce the interaction of COP-I with STING, occurring via the cargo receptor Surfeit 4 (3, 5, 68), leading to defective retrieval of STING from the Golgi and, hence, amplified STING activity (3, 4, 68, 94, 95). These mutations most frequently localize to the N-terminus WD40 domain of COPA (96), a region critical to dilysine motif–mediated cargo binding of cell constituents for intracellular transport (reviewed in ref. 97). Recently, mutations in the C-terminal domain of COPA, predicted to alter COP-I integrity, were reported to cause an inflammatory phenotype manifesting as complex cases of autoinflammation and autoimmunity (98). Some patients experienced alveolar hemorrhage, one in the neonatal period, but patients also had some features apparently distinct to classical COPA syndrome (98).

Individuals with COPA syndrome usually demonstrate elevated ISG expression in PBMCs (4, 84) and lung fibroblasts (3). Heightened IFN-I production in response to COPA syndrome–associated mutations, compared with WT COPA, has been found to be STING dependent (4). These experiments utilized cotransfection of STING and COPA plasmids (with and without COPA syndrome mutations) into HEK cells, which are known to not express STING (4). COPA protein expression is preserved in cells from patients with pathogenic *COPA* mutations (5), indicating a dominant-negative effect of mutant COPA (4, 68). Moreover, knockdown of COPA or overexpression of COPA mutations in cell lines increases ER stress and promotes the production of Th17-inducing cytokines (5). Importantly, the common HAQ STING haplotype was found to ameliorate the heightened STING signaling associated with disease-related *COPA* mutations, attributed to HAQ STING reducing COPA-dependent STING activation (99). In that study, all nine asymptomatic individuals with *COPA* mutations had the HAQ haplotype, and the investigators concluded that this fully explained the recognized clinical nonpenetrance seen in COPA syndrome (99). More recently, however, five asymptomatic individuals with *COPA* mutations were found not to have the HAQ haplotype (100), implying that factors in addition to HAQ status are involved in influencing penetrance of COPA syndrome (Table 2). This raises potentially interesting avenues for further study.

Several mouse models have been developed to study the effects of *COPA* mutations. *Copa^E241K/+^* mice, which harbor a heterozygous pathogenic mutation causing COPA syndrome in humans, spontaneously developed ILD when aged 10–11 months (86). This recapitulates the ILD observed in COPA syndrome patients, with cellular bronchiolitis, germinal center formation, and CD4^+^ T cell infiltrates. No joint inflammation or alveolar hemorrhage was observed (86). *Copa^E241K/+^* mice displayed an increased frequency of splenic effector T cells capable of producing Th17-associated cytokines, and adoptive transfer of *Copa^E241K/+^* T cells into immunodeficient mice was sufficient to induce some lung inflammation, indicating a role for T cells in the pathogenesis of lung inflammation in the *Copa^E241K/+^* model (86). The T cell phenotype in *Copa^E241K/+^* mice has been shown to relate to impaired T cell negative selection in the thymus driven by STING (3, 101). Crossing *Copa^E241K/+^* mice into a *Sting*^–/–^ line rescued embryonic lethality of *Copa* homozygous mutant mice, further strengthening the link to STING activity (3).

Lung inflammation was similarly described in another COPA mouse model, *Copa^V242G/+^*, which had evidence of lymphocytic and macrophage infiltration and lymphoid follicles in the lung as well as changes in T cell composition (84). Interestingly, *Copa^V242G/+^* bone marrow–derived DCs did not display elevated baseline ISG expression; instead, potentiated responses following stimulation with a STING agonist were noted, implying that an initial stimulus may be required to elicit heightened signaling (84). In addition, binding of COP-I to STING did not seem to be reduced, in contrast with other pathogenic mutations, such as p.(E241K) (84). Further study of these models are necessary to fully elucidate the pathogenesis of lung disease in COPA syndrome.

## Current treatment strategies for SAVI and COPA syndrome

It can be difficult to draw firm conclusions regarding treatment efficacy in ultrarare conditions such as SAVI and COPA syndrome, but overall, generalized immunosuppression and antifibrotic treatments do not seem to be effective, particularly in the treatment of progressive lung disease (62, 84). However, combinatorial immunosuppressive regimens have been reported to ameliorate pulmonary dysfunction in some cases (88, 102), and further collation of clinical outcome data will be beneficial, including longer-term outcomes in the limited number of lung transplantations that have taken place (62, 84). At present, the use of JAK inhibitors, such as baricitinib, appear to be the most therapeutically favorable option, leading to improvement in features such as arthritis in some COPA patients and stabilization, or even improvement, of lung disease in some individuals with SAVI or COPA syndrome (4, 63, 74, 84, 86, 103). In a large European cohort, JAK inhibition was found to have some clinical effect in two-thirds of patients with COPA syndrome, but progression of disease, including lung disease, still occurred in the remaining patients (74). JAK inhibitors appear to work best when administered early in the management of lung disease, rather than once fibrosis is established (62).

It is possible that future development of novel therapeutic approaches targeting the STING pathway, or downstream sequelae of STING activation, could eventually be an option for treating SAVI or COPA syndrome, since pharmacological STING inhibition presents clear therapeutic opportunities for monogenic lung diseases such as SAVI and COPA where ILD is linked to STING hyperactivity. Efforts to develop STING antagonists to inhibit STING-driven inflammatory disease are ongoing, but these approaches are predominantly at the preclinical research stage at present (8, 104). Table 3 depicts different approaches to limit STING activity. Further understanding of the actions of STING in the lung, particularly in different cell types, is likely to be beneficial to the ongoing research in this area.

## Does STING play a role in other ILDs?

The seemingly clear association of hyperactivation of the STING pathway with ILD in SAVI and COPA syndrome raises the question of whether STING is relevant to the pathogenesis of more prevalent forms of ILD. One possible route for STING activation could be the release of dsDNA from damaged cells in the lung, which could induce cGAS/STING pathway activation (105). Indeed, Benmerzoug et al. found higher levels of dsDNA in BAL fluid from patients with the ILD silicosis compared with healthy controls (106), and the same researchers also found that *Sting1^–/–^* mice had less severe silica-induced lung inflammation than WT animals (106). Additionally, airway epithelial cells and fibroblasts from IPF patients have been shown to be prone to senescence due to abnormal activation of the cGAS/STING pathway (107, 108). Roles for STING in the susceptibility to lung fibrosis following viral infection, and in the immune response to cigarette smoke–induced cell damage, have also been reported in other mouse models (109, 110).

A common mouse model of ILD involves the administration of the chemotherapy agent bleomycin (which causes DNA breakage) to the lungs. This induces a period of inflammation relating to acute cellular damage (e.g., of alveolar epithelial cells) and is associated with an influx of immune cells for approximately 9 days (111, 112). Lung fibrosis usually develops by day 14, peaks around days 21–28, and may spontaneously resolve thereafter (112). There is considerable variability across mouse strains in terms of susceptibility to experimental pulmonary fibrosis. The reasons for this include differences in the ability to metabolize bleomycin, varied immune responses during the initial inflammatory phase, and reduced TGF-β gene and protein expression, collectively highlighting the crucial coupling of inflammation and fibrosis in the pathogenesis of certain ILDs (113). Extracellular dsDNA has been shown to accumulate in BAL fluid following bleomycin administration, leading to STING overexpression in the lung (114–117). Furthermore, increased phosphorylation of STING, TBK1, and IRF3 was found at day 7 following bleomycin administration, indicative of STING pathway activation (115). Zhang et al. found *Sting^–/–^* mice had reduced lung fibrosis at day 28 after one intratracheal dose of bleomycin compared with *Sting^+/+^* animals (118). Intratracheal administration of cGAMP also induced lung fibrosis and a STING-mediated senescence response (118). Taken together, these studies suggest a profibrotic role for STING following bleomycin administration.

Importantly, however, a role for STING activity in mouse models of ILD is not universal. Savigny et al. found that *Sting^–/–^* mice manifested more severe lung fibrosis than *Sting^+/+^* mice at day 14 following bleomycin administration (117). Furthermore, a different study found that mice lacking IFNAR1 (a key component of the IFN-I receptor complex) had increased bleomycin-induced lung inflammation and fibrosis at day 14 compared with WT counterparts (119). It is possible that these differences in experimental outcomes may indicate distinct roles of STING at different stages of bleomycin-driven pathology, relate to different commensal microbiota populations due to various housing conditions, or denote differential effects of STING in different settings. Indeed, while the role for STING in promoting inflammation in a variety of diseases is well documented, emerging evidence suggests that the effects of STING activity on the immune response are more nuanced and that in some contexts, STING activation may instead lead to immune regulation (59, 120). This is an important area of interest in the field of cancer immunology and is likely to be context dependent (121). Overall, further research into the role of STING in the pathogenesis of more common ILDs is still required.

## Conclusion

Genetic evidence implicates overactive STING signaling in ILD pathogenesis in the monogenic conditions SAVI and COPA syndrome. Exactly how STING activity leads to ILD in these ultrarare diseases remains an area of active study that will inform understanding about the biological processes involved in lung inflammation. Some, but not all, human and mouse studies have linked STING activity to the initiation and/or progression of certain types of ILD, but ILDs comprise a broad range of disease subtypes, and it is likely that the functional outcomes of STING activation may vary in different disease settings. Further mechanistic research into the role of STING in ILD pathogenesis is warranted to explore the possibility of STING-centered therapeutics for ILDs.

## Conflict of interest

The authors have declared that no conflict of interest exists.

## Funding support

- Wellcome PhD Clinical Training Fellowship (PPV).
- LifeArc Centre for Rare Respiratory Diseases (GZ and KJM).
- Medical Research Council Clinician Scientist Fellowship (grant MR/W02487X/1 to KJM).
- LifeArc Rare Respiratory Diseases Centre (KJM).

## Figures and Tables

**Table 1:**
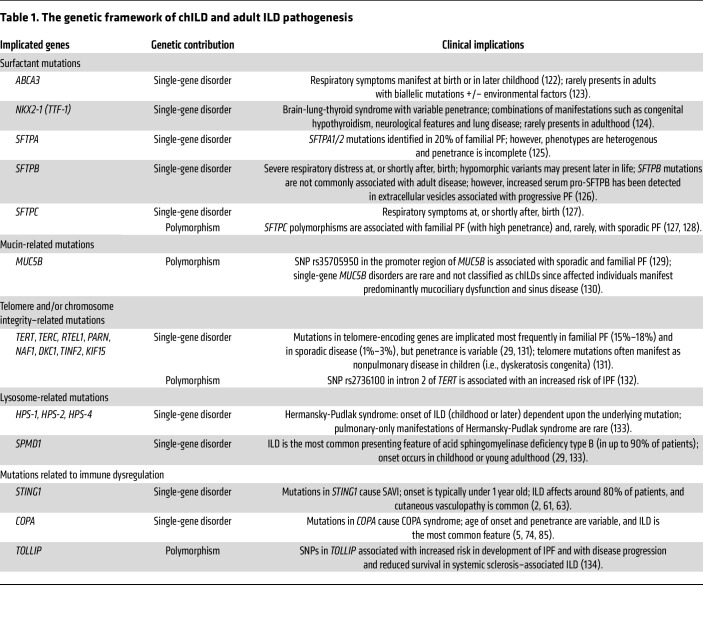
The genetic framework of chILD and adult ILD pathogenesis

**Table 2:**
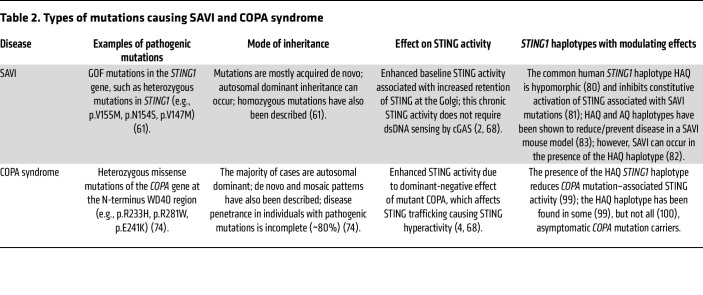
Types of mutations causing SAVI and COPA syndrome

**Table 3:**
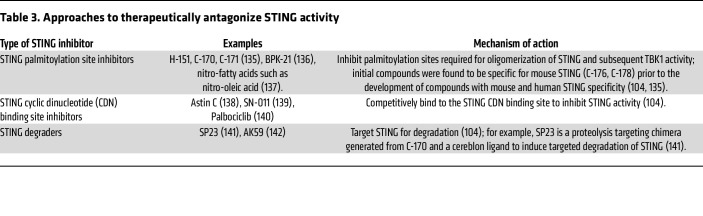
Approaches to therapeutically antagonize STING activity

## Version 1 06/01/2026

Electronic publication
